# The Pan-Immune-Inflammation-Value Predicts the Survival of Patients with Human Epidermal Growth Factor Receptor 2 (HER2)—Positive Advanced Breast Cancer Treated with First-Line Taxane-Trastuzumab-Pertuzumab

**DOI:** 10.3390/cancers13081964

**Published:** 2021-04-19

**Authors:** Francesca Ligorio, Giovanni Fucà, Emma Zattarin, Riccardo Lobefaro, Luca Zambelli, Rita Leporati, Carmen Rea, Gabriella Mariani, Giulia V. Bianchi, Giuseppe Capri, Filippo de Braud, Claudio Vernieri

**Affiliations:** 1 Medical Oncology Unit, Fondazione IRCCS Istituto Nazionale dei Tumori, Via Venezian 1, 20133 Milan, Italy; giovanni.fuca@istitutotumori.mi.it (G.F.); emma.zattarin@istitutotumori.mi.it (E.Z.); riccardo.lobefaro@istitutotumori.mi.it (R.L.); luca.zambelli@istitutotumori.mi.it (L.Z.); rita.leporati@istitutotumori.mi.it (R.L.); carmen.rea@istitutotumori.mi.it (C.R.); gabriella.mariani@istitutotumori.mi.it (G.M.); Giulia.bianchi@istitutotumori.mi.it (G.V.B.); Giuseppe.capri@istitutotumori.mi.it (G.C.); Filippo.debraud@istitutotumori.mi.it (F.d.B.); 2 Department of Oncology and Hemato-Oncology, University of Milan, 20133 Milan, Italy; 3 Fondazione Istituto FIRC di Oncologia Molecolare (IFOM), Via Adamello 16, 20139 Milan, Italy

**Keywords:** HER2-positive breast cancer, trastuzumab + pertuzumab, PIV, NLR, MLR, PLR

## Abstract

**Simple Summary:**

Although taxane-trastuzumab-pertuzumab combinations in the first-line treatment setting significantly improved clinical outcomes in patients with Human Epidermal growth factor Receptor 2 positive (HER2+) advanced breast cancer (aBC), their clinical efficacy is highly heterogeneous, and reliable biomarkers of benefit from this treatment are lacking. Different peripheral blood parameters have emerged as prognostic biomarkers in breast cancer, but their predictive role in HER2+ BC patients receiving dual anti-HER2 blockade remains unclear. In this work, we identified the Pan-Immune-Inflammatory Value (PIV), a recently defined parameter, taking into account peripheral blood neutrophil, platelet, monocyte and lymphocyte counts, as an independent predictor of worse OS in patients with HER2+ aBC receiving first line trastuzumab-pertuzumab biochemotherapy. The PIV outperforms other well-known peripheral blood parameters, thus potentially representing a new tool to improve the prognostic stratification of HER2+ aBC patients in a first-line treatment setting.

**Abstract:**

Different peripheral blood parameters have emerged as prognostic biomarkers in breast cancer (BC), but their predictive role in Human Epidermal growth factor Receptor 2 positive (HER2+) advanced BC (aBC) patients receiving dual anti-HER2 blockade remains unclear. We evaluated the impact of the Pan-Immune-Inflammatory Value (PIV), defined as the product of peripheral blood neutrophil, platelet, and monocyte counts divided by lymphocyte counts, on the prognosis of HER2+ aBC patients treated with first line trastuzumab-pertuzumab-based biochemotherapy. We also evaluated the association between the neutrophil-to-lymphocyte ratio (NLR), the platelet-to-lymphocyte ratio (PLR), and the monocyte to lymphocyte ratio (MLR) and clinical outcomes. Cox regression models were used to estimate the impact of these variables, as well as of other clinically relevant covariates, on patient survival. We included 57 HER2+ aBC patients treated with taxane-trastuzumab-pertuzumab in our Institution. High baseline MLR, PLR, and PIV were similarly predictive of worse PFS at univariate analysis, but only high PIV was associated with a trend toward worse PFS at multivariable analysis. Regarding OS, both high PIV and MLR were associated with significantly worse patient survival at univariate analysis, but only the PIV was statistically significantly associated with worse overall survival at multivariable analysis (HR 7.96; 95% CI: 2.18–29.09). Our study reveals the PIV as a new and potent predictor of OS in HER2+ aBC patients treated with first line trastuzumab-pertuzumab-containing biochemotherapy. Prospective studies are needed to validate this new prognostic parameter in HER2+ aBC.

## 1. Introduction

The overexpression of Human Epidermal Growth Factor Receptor 2 (HER2) is detected in approximately 15–20% of breast cancers (BCs), and is typically associated with high biological and clinical disease aggressiveness [1,2]. Trastuzumab and pertuzumab are humanized monoclonal antibodies that inhibit the oncogenic HER2 protein either directly (trastuzumab) or by inhibiting its dimerization with HER3 (pertuzumab) [3,4,5]. When compared with single trastuzumab or pertuzumab treatments, the trastuzumab-pertuzumab combination induces a more complete suppression of HER2 signaling, thus resulting in synergistic anticancer effects against HER2 positive (HER2+) BC cells [6]. Of note, part of trastuzumab and pertuzumab antitumor effects are mediated by immune system activation against cancer cells, a phenomenon known as antibody-dependent cytotoxicity (ADCC) [7].

In the phase III trial CLEOPATRA, adding pertuzumab to trastuzumab plus docetaxel in the first line setting significantly prolonged both progression free survival (PFS) and overall survival (OS) of patients with HER2+ advanced BC (aBC), with a median OS of more than 56 months in pertuzumab-treated patients [8]. However, despite this dramatic improvement, approximately 20% of HER2+ aBC patients receiving dual anti-HER2 blockade undergo precocious disease progression (i.e., within 6 months after treatment initiation) as a result of primary tumor resistance [9]. A biomarker analysis of the CLEOPATRA trial demonstrated that high expression levels of HER2 and HER3, as evaluated through quantitative mRNA assessment, or wild-type PIK3CA gene, are associated with significantly better prognosis in this clinical context [10]. However, the clinical impact of this work is limited by the fact that these biomarkers are not commonly evaluated in several cancer centers; in addition, no real-life studies have prospectively validated these results. Therefore, the identification of new, cheap, and easily assessable biomarkers capable of stratifying patients based on their benefit from trastuzumab-pertuzumab biochemotherapy, as well as in terms of long-term clinical outcomes, is of primary importance.

The activation of anti-tumor adaptive T cell immune responses has emerged as both prognostic and predictive in HER2+ BC patients. In particular, high tumor infiltrating lymphocytes (TILs) correlated with higher rates of pathological complete response in the neoadjuvant treatment setting [11,12,13], as well as with better OS in aBC patients receiving dual anti-HER2 blockade [14]. Given the established immune-mediated effects of both trastuzumab and pertuzumab, the benefits of these drugs in BC patients may at least in part depend on the activation status of the immune system and the balance between inflammatory pro-tumor and anti-tumor immune populations. Recent studies highlighted the prognostic role of easily measurable blood parameters that reflect systemic inflammation, such as the neutrophil-to-lymphocyte ratio (NLR) [15], the platelet-to-lymphocyte ratio (PLR) [16], and the monocyte to lymphocyte ratio (MLR) [17] in different solid malignancies. Furthermore, several large studies showed an association between high NLR and/or PLR and poor clinical outcomes in heterogeneous BC patient populations [18,19,20,21], but these studies did not report subgroup analyses according to specific BC subtypes or to the type of therapy received. Regarding specific BC subgroups, a relationship between low NLR and good prognosis was reported in patients with the triple negative (TNBC) subtype [22,23,24] and in patients with HER2+ aBC receiving TDM-1 [25]. However, the role of these biomarkers in HER2+ aBC patients treated with dual anti-HER2 blockade remains unclear.

Recently, a new biomarker that takes into account all routinely assessed blood cell populations reflecting systemic inflammation and immunity, i.e., neutrophils, monocytes, lymphocytes, and platelets, has been proposed as a stronger and more reliable predictor of clinical outcomes in patients with advanced colorectal cancer [26]. This biomarker, which was defined as Pan-Immune-Inflammation Value (PIV), was strongly associated with both PFS and OS in metastatic colorectal cancer patients receiving first-line biochemotherapy, and it outperformed other established immune biomarkers in predicting patient outcome.

Here, we investigated the potential role of the PIV as a predictive and/or prognostic biomarker in HER2+ aBC patients treated with first line trastuzumab and pertuzumab.

## 2. Materials and Methods

### 2.1. Study Setting

This was a retrospective, monocentric study in HER2+ aBC patients who initiated first-line taxane-trastuzumab-pertuzumab biochemotherapy between April 2014 and September 2020 at Fondazione IRCCS Istituto Nazionale dei Tumori (Milan, Italy).

The eligibility criteria were: (1) age ≥ 18 years; (2) pathologically or cytologically confirmed diagnosis of unresectable, locally recurrent or metastatic HER2+ aBC, as defined as an immunohistochemistry (IHC) score for HER2 of 3+, or an equivocal IHC score (2+) with in situ hybridization (ISH) indicating *HER2* gene amplification; (3) treatment with one of the following chemotherapy plus anti-HER2 therapy schedules: every-three-week docetaxel plus trastuzumab plus pertuzumab, or weekly paclitaxel plus every-three-week trastuzumab plus pertuzumab; (4) availability of baseline (pre-treatment) absolute peripheral blood neutrophil, monocyte, lymphocyte, and platelet counts; (5) available information about previous treatment(s) for limited-stage disease; (6) available information on the date of disease progression and patient death; (7) absence of acute infections or documented bone marrow infiltration at the time of peripheral blood cell count assessment. This study was approved by Local Ethics Committee of Fondazione IRCCS Istituto Nazionale dei Tumori (INT 170/20). Patient data were collected according to the ethical principles for medical research involving human subjects adopted in the Declaration of Helsinki. Patients who were alive at the time of data collection and/or analysis signed an informed consent for the use of their personal data for research purposes.

The data that support the findings of this study are available from the corresponding authors upon reasonable request.

### 2.2. Objectives

The objective of the study was to investigate the association between baseline peripheral blood parameters (i.e., PIV and, secondarily, NLR, PLR, and MLR) and clinical outcomes in terms of PFS, as defined as the time between treatment initiation and disease progression or patient death from any cause, and OS, as defined as the time between treatment initiation and death from any cause.

Tumor response was evaluated every three treatment cycles (i.e., approximately every two months), but disease re-evaluation was anticipated in those patients with deteriorating symptoms or other signs suggestive of progressive disease. The tumor response was assessed according to the Response Evaluation Criteria in Solid Tumors (RECIST 1.1).

All patients were followed up until death, loss of contact, or time of data lock (1 February 2021).

### 2.3. Evaluation of Biomarkers

We collected data on absolute counts of peripheral blood neutrophils, lymphocytes, platelets, and monocytes. The parameters should have been measured within one month before treatment initiation, provided that no concomitant antineoplastic therapies were administered at the time of blood collection. Patients whose blood parameters were measured more than one month before treatment initiation, or after having received the first dose of the study treatment, were excluded from the analysis. From blood cell counts we calculated the following parameters: (a) PIV by multiplying neutrophil count (10^3^/mmc) by platelet count (10^3^/mmc) and monocyte count (10^3^/mmc) and, finally, dividing the result of this product by lymphocyte count (10^3^/mmc) [26]; (b) NLR by dividing neutrophil count (10^3^/mmc) by lymphocyte count (10^3^/mmc); (c) PLR by dividing platelet count (10^3^/mmc) by lymphocyte count (10^3^/mmc); (d) MLR by dividing monocyte (10^3^/mmc) by lymphocyte count (10^3^/mmc). The cut-off points to define patients with high or low PIV, NLR, PLR, or MLR were defined as median values of these parameters in the clinical cohort.

### 2.4. Statistical Analysis

Patient characteristics were analyzed by descriptive statistics. Two sided χ2 or Fisher’s exact tests were used to evaluate the association between categorical variables, while the Wilcoxon–Mann–Whitney test was used for continuous variables. PFS and OS were represented through to the Kaplan–Meier method, and the log-rank test was used to compare the survival distributions of different patient populations. The impact of known prognostic factors on PFS and OS was first assessed at univariate analysis. Covariates associated with the risk of progression (*p* < 0.1) were then included in a multivariable Cox proportional hazard model to assess their independent association with survival. The following clinically relevant covariates were evaluated: age (≥50 years vs. <50 years), hormone receptor (HR) status (positive vs. negative), *de novo* advanced disease (yes vs. no); number of metastatic sites (1–2 vs. >2); presence of visceral metastases (yes vs. no); presence of brain metastases (yes vs. no); previous exposure to trastuzumab (yes vs. no); previous exposure to taxanes (yes vs. no); previous exposure to anthracyclines (yes vs. no). Statistical analyses were performed using R (version 3.6.1, https://cran.r-project.org/bin/macosx/ accessed on 11 March 2021) and R Studio (version 1.2.5042). A *p*-value of 0.05 was chosen as the threshold level for statistical significance.

## 3. Results

### 3.1. Patient Characteristics

The characteristics of evaluated patients are described in Table 1. Among 57 patients included in the analysis, median baseline PIV was 285 (range 0–4249), median baseline PLR was 159 (range 24–1347), median baseline MLR was 0.3 (range, 0–1) and median baseline NLR was 3.0 (range, 0.9–14.6). Compared to patients with low PIV, a higher proportion of patients with high PIV had brain or visceral metastases, while there was no significant association between PIV category and other patient or disease characteristics, such as age, de novo vs. recurrent advanced BC, HR status, number of metastatic sites, previous treatment with anthracyclines, taxanes, or trastuzumab (Table S1). We found a moderate, positive correlation between NLR and PLR (R = 0.61), as well as between NLR and MLR (R = 0.57), while there was a weak positive correlation between PLR and MLR (R = 0.25). We also found a moderate positive correlation between PIV and NLR (R = 0.51), as well as between PIV and MLR (R = 0.64), while PIV and PLR (R = 0.32) were weakly correlated (Table S2 and Figure S1).

### 3.2. Impact of Peripheral Blood Parameters on PFS

**Figure 1:**
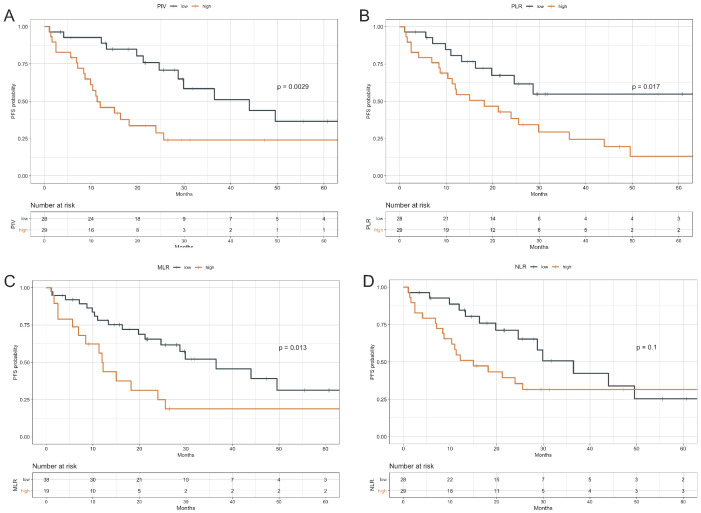
Progression Free Survival according to baseline PIV, PLR, MLR and NLR values. Kaplan–Meier curves representing patient PFS according to baseline PIV (**A**), PLR (**B**), MLR (**C**), and NLR (**D**) categories. The median value of each parameter was used as a threshold for the definition of the parameter categories (high vs. low). PFS: Progression-free survival; PIV: Pan-Immune -Inflammation Value; MLR: monocyte to lymphocyte ratio; PLR: platelet to lymphocyte ratio. NLR: neutrophil to lymphocyte ratio. The I symbol indicates patients censored at the time of data cut off and analysis.

With a median follow-up of 36.6 months (95% CI: 31.1–58.5), a total of 32 tumor progression events were detected, with a median PFS of 24.6 months in the study population (95% CI, 16.3–49.6). PFS was significantly longer in patients with low baseline PIV as compared to high PIV (median PFS: 44 months, 95% CI 28.72–NR vs. 11.3 months, 95% CI 8.72–25.6, *p* = 0.003) (Figure 1A). High PLR or MLR were also similarly associated with worse PFS. Regarding PLR, median PFS was not reached (95% CI: 24.6–NR) in patients with low baseline PLR, while it was 18.2 months (95% CI: 11.3–36.5) in patients with high PLR (*p* = 0.017) (Figure 1B). As for MLR, median PFS was 36.5 months (95% CI, 24.64–NR) in patients with low MLR when compared with 12 months (95% CI: 6.91–NR) in patients with high MLR (*p* = 0.013) (Figure 1C). By contrast, high NLR was associated with only a trend toward worse PFS (median PFS: 36.5 months (95% CI, 24.6–NR) in patients with low NLR; 15.0 months (95% CI: 10.4–NR) in patients with high NLR (*p* = 0.1) (Figure 1D). When we evaluated the other immune-related parameters (NLR, MLR or PLR) at multivariable analysis, none of them was found to be independently associated with patient PFS (Table S3). Of note, patients achieving a long disease control (i.e., with a PFS of at least 24 months, which was the median PFS in our patient population) had significantly lower PIV values, both when evaluated as a continuous variable (median PIV: 205 (range 65–2635) in patients with PFS ≥ 24 months vs. 451 (range: 0–4249) in patients with PFS < 24 months, *p* = 0.018, Kruskal-Wallis test) (Figure S2) and as dichotomous variable (Table S5) when compared to patients with a shorter PFS.

At univariate analysis, other factors associated with worse PFS were a higher number of metastatic sites, the presence of visceral metastases, and prior (neo)adjuvant therapy with trastuzumab. (Table 2, upper panel). Since we found a positive correlation between NLR and PLR, NLR and MLR, and PLR and MLR (Table S2), the PIV, which incorporates the potential prognostic impact of all potentially relevant parameters, was the only immunological covariate initially evaluated in a multivariable model including other clinically relevant variables. As shown in Table 2 (lower panel), a higher number of metastatic sites (HR 3.78, 95% CI, 1.50– 9.52, *p* = 0.005) and prior therapy with trastuzumab (HR 4.29, 95% CI 1.85–9.96, *p* < 0.001) were independently associated with worse PFS, while high PIV did not show a statistically significant and independent association with worse PFS (HR 2.34, 95% CI 0.99–5.51; *p* = 0.052) (Table 2, lower panel). When we evaluated the other immune-related parameters (NLR, MLR or PLR) at multivariable analysis, none of them was found to be independently associated with patient PFS (Table S3). Of note, patients achieving a long disease control (i.e., with a PFS of at least 24 months, which was the median PFS in our patient population) had significantly lower PIV values, both when evaluated as a continuous variable (median PIV: 205 (range 65–2635) in patients with PFS ≥ 24 months vs. 451 (range: 0–4249) in patients with PFS < 24 months, *p* = 0.018, Kruskal-Wallis test) (Figure S2) and as dichotomous variable (Table S5) when compared to patients with a shorter PFS.

### 3.3. Impact of Peripheral Blood Parameters on OS

**Figure 2:**
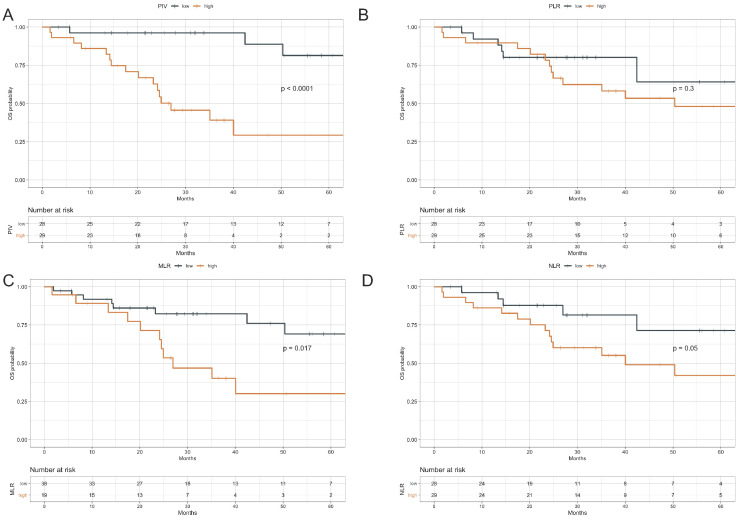
Overall Survival according to PIV, PLR, MLR, and NLR. Kaplan–Meier curves representing patient OS according to baseline PIV (**A**), PLR (**B**), MLR (**C**) and NLR (**D**) categories. The median value of each parameter was used as a threshold for the definition of the parameter categories (high vs. low). OS: Overall survival; PIV: Pan-Immune-Inflammation Value; MLR: monocyte to lymphocyte ratio; PLR: platelet to lymphocyte ratio. NLR: neutrophil to lymphocyte ratio. The I symbol indicates patients censored at the time of data cut off and analysis.

During the study follow-up, 20 death events occurred, with a median OS of 64.6 months in the study population (95% CI, 40–NR). Higher PIV and MLR were associated with significantly lower OS (log rank test: *p* < 0.0001 and *p* = 0.017, respectively), while higher NLR was associated with only a trend toward worse OS (*p* = 0.05). On the contrary, we found no significant association between PLR and OS (*p* = 0.3). Specifically, in high-PIV vs. low-PIV patients 3-year OS rate was 39.1% (95%CI: 23.1–66.2) vs. 96.2% (95%CI: 89.0–100.0); in high-PLR vs. low-PLR patients 3-year OS rate was 58.2% (95% CI: 41.9–80.8) vs. 80.1 (95% CI: 65.9–97.4); while in high MLR vs. low MLR patients 3-year OS rate was 82.3 (95% CI: 70.3–96.4) vs. 40.1% (95% CI: 22.1–72.9) (Figure 2).

Other factors associated with worse OS at univariate analysis were: a higher number of metastatic sites, the presence of visceral metastases, and the presence of brain metastases. (Table 3, upper panel). In the multivariable model that included PIV and these clinical variables, the PIV was the only covariate independently associated with worse OS (7.96, 95% CI 2.18–29.09; *p* = 0.002) (Table 3 lower panel). Multivariable models including the same clinical variables and NLR, MLR, or PLR showed no association between each of immune-related variables and OS (Table S4).

## 4. Discussion

In the present study, we showed for the first time the negative prognostic impact of high PIV in HER2+ aBC patients treated with first line trastuzumab-pertuzumab-containing biochemotherapy.

Different peripheral blood cell populations can reflect systemic and intratumor inflammatory/immune system status. Peripheral blood lymphocyte counts can mirror the activation status of adaptive immunity, while low lymphocyte counts are commonly found in conditions of poor nutrition and cachexia [27,28,29]. On the other hand, monocytes represent a subpopulation with an emerging role in cancer progression, as they are the progenitor cells of tumor-associated macrophages (TAMs), which suppress intratumor populations of effector cells, such as cytotoxic T cells and Natural Killer cells [30,31,32]. High neutrophils might reflect a state of systemic inflammation or immune suppression [33] and, similarly to TAMs, tumor-associated neutrophils (TANs) are capable of inhibiting CD8+ T cell antitumor activity [34]. Finally, a high platelet count, which is associated with systemic inflammation, correlates with tumor metastatic potential, and is a recognized negative prognostic factor in many types of human neoplasms [35]. Published works have demonstrated the prognostic impact of parameters combining different blood cell populations in patients with several cancer types [16,36,37], and their stronger association with patient outcomes when compared to individual blood cell counts [22,38]. Regarding BC, high NLR or high PLR have been associated with worse patient prognosis in specific clinical subsets. For instance, we previously showed that high NLR and PLR are associated with worse PFS and OS in patients with advanced triple-negative breast cancer (TNBC) treated with platinum-containing chemotherapy, but not in patients with advanced hormone receptor-positive breast cancer [22]. Notably, NLR has been shown to positively correlate with the number of blood myeloid derived suppressor cells (MDSCs) in a cohort of BC patients, highlighting its role as a biomarker of immunosuppressive state [39].

Although established evidence indicates that the antitumor effects of trastuzumab are at least in part mediated by immune system activation and ADCC, no clinical evidence exists to support the prognostic role of peripheral blood immune cell populations in HER2+ aBC patients treated with first line trastuzumab-pertuzumab-based combinations. A subgroup analysis of a study that included early-stage BC (eBC) patients treated with curative surgery did not show any prognostic impact of NLR, PLR or MLR in the subset of patients with HER2+ BC [38]. However, only 37 HER2+ BC patients were included in the analysis, with a consequently limited number of reported progression and death events. Similarly, Ulas et al. did not find any significant association between NLR or PLR and clinical outcomes in a cohort of HER2+ eBC patients receiving adjuvant trastuzumab [40]. Several explanations could justify the discrepancies between these findings and the results of our study. Firstly, previously published studies might have been underpowered to detect any DFS or OS difference, as suggested by the fact that these works reported a trend, yet not statistically significant, towards an association between high NLR and shorter median DFS. Alternatively, the prognostic impact of blood parameters accounting for systemic and intratumor inflammation/immunity might be more strongly associated with the prognosis of HER2+ BC patients with advanced disease, which is typically characterized by more severe alterations in the number or activation status of specific immune cell populations [41]. In this view, results of the recent study by Imamura et al. [25], who found a significant association between high NLR/PLR and worse PFS and OS in HER2+ aBC patients treated with second line TDM-1, are in line with the findings of our study.

More recently, the PIV has been proposed as a new and more potent predictor of clinical outcomes in cancer patients [26]. PIV showed a strong association with PFS and OS in patients with advanced colorectal cancer, and the prognostic value of the PIV was stronger than that of other well-established immune-inflammatory biomarkers (e.g., NLR). When compared with individual blood cell parameters, the PIV might be able to more comprehensively capture the complexity of the immune contexture and its numerous components, each of which might reflect and regulate different aspects of antitumor immunity [26]. In line with these previously published data in advanced colorectal cancer, we found that high PIV is independently associated with worse patient OS in in HER2+ aBC, while high NRL, MLR, or PLR are not. A trend toward an independent association between high PIV and worse PFS was observed at multivariable analysis (*p* = 0.052), even if a formal statistical significance was not reached. Nonetheless, median PIV scores were significantly lower in patients achieving a long disease control, thus suggesting that this parameter may be useful to select a patient population with a particularly good prognosis and needs further investigation.

The fact that the observed impact of the PIV on the OS of HER2+ aBC patients treated with first line trastuzumab + pertuzumab containing biochemotherapy does not correspond to a similarly strong impact on patient PFS could be explained by the hypothesis that the PIV might be more generally prognostic, rather than specifically predictive of benefit to double anti-HER2 blockade, in this clinical context. Indeed, by incorporating neutrophils, platelets, monocytes, and lymphocytes, the PIV represents a composite parameter that comprehensively defines the status of systemic inflammation and immune system activation in HER2+ BC patients. For this reason, the PIV could amplify the contribution of individual cell counts on patient prognosis, and in particular the fact that even a slight increase of individual immune cell populations individually associated with clinical outcomes, such as monocytes, platelets and neutrophils, might have a multiplicative effect in affecting long-term patient survival; in particular, high PIV scores might reflect a systemic state of immunosuppression characterized by different combinations of peripheral blood immune cell subpopulations in the same high-risk population.

The strengths of this study consist if: (1) its monocentric design, which guarantees homogeneous patient treatment and follow-up, as well as a more reproducible collection of laboratory data; (2) the fact that the evaluated blood parameters are cheap and easily assessable biomarkers that can be calculated from routinely collected blood cell count data; and (3) the novelty regarding the independent association between high PIV and worse OS. The weaknesses of our study are the relatively small number of patients included in the analysis, the retrospective nature of the investigation, the short follow-up time, and the lack of analysis of TILs, which were previously found to predict the clinical outcomes of advanced HER2+ BC patients enrolled in the CLEOPATRA trial, and which could have allowed us to correlate the evaluated peripheral blood parameters to the activation status of both systemic and intratumor immune compartments.

## 5. Conclusions

In conclusion, our work identifies PIV as a systemic immune score that is strongly associated with OS in HER2+ aBC patients receiving first-line taxane-trastuzumab-pertuzumab biochemotherapy. Of note, PIV outperforms NLR, PLR, and MLR in predicting OS. If validated in prospective studies, the PIV could represent a new tool to improve the prognostic stratification of HER2+ aBC patients treated with dual HER2 blockade, and in particular for the identification of patients with especially long expected OS.

## Figures and Tables

**Table 1:** Patient and disease characteristics.

Characteristic	Total (*n* = 57)
AGE	
Median (Range)	53 (26–78)
<50	19 (33.3%)
≥50	38 (66.7%)
*De novo* metastatic disease	
no	34 (59.6%)
yes	23 (40.4%)
Number of metastatic sites	
≤2	30 (52.6%)
>2	27 (47.4%)
Visceral metastases	
no	27 (47.4%)
yes	30 (52.6%)
Brain metastases	
no	49 (86.0%)
yes	8 (14.0%)
HR status *	
negative	20 (35.1%)
positive	37 (64.9%)
Previous trastuzumab	
no	35 (61.4%)
yes	22 (38.6%)
Previous anthracyclines	
no	30 (52.6%)
yes	27 (47.4%)
Previous taxanes	
no	31 (54.4%)
yes	26 (45.6%)
Type of chemotherapy regimen	
Docetaxel	44 (77.2%)
Paclitaxel	13 (22.8%)
PIV	
Median (range)	285 (0–4249)
PLR	
Median (range)	159 (24–1347)
MLR	
Median (range)	0.3 (0.0–1.0)
NLR	
Median (range)	3.0 (0.9–14.6)

Data are presented as *n* (%) except where otherwise noted. * Defined positive if >1% of tumor cells express hormone receptors. Abbreviations: HR, hormone receptors; PIV: Pan-Immune -Inflammation Value; PLR: platelet to lymphocyte ratio; MLR: monocyte to lymphocyte ratio; NLR: neutrophil to lymphocyte ratio.

**Table 2:** Univariate and multivariable Cox proportional hazards model for PFS.

Univariate Analysis	HR (95% CI)	*p*
AGE		
per 1 year	1.01 (0.98–1.04)	0.550
≥50 vs. <50	1.49 (0.70–3.18)	0.307
HR status *		
Positive vs. negative	0.94 (0.45–1.96)	0.873
*De novo* advanced disease		
yes vs. no	0.73 (0.35–1.51)	0.395
Number of metastatic sites		
≥3 vs. <3	2.36 (1.13–4.93)	**0.022**
Visceral metastases		
yes vs. no	2.14 (1.03–4.44)	**0.041**
Brain metastases		
yes vs. no	1.96 (0.74–5.17)	0.175
Previous trastuzumab		
yes vs. no	1.92 (0.96–3.86)	**0.067**
Previous anthracyclines		
yes vs. no	1.05 (0.52–2.11)	0.887
Previous taxanes		
yes vs. no	1.69 (0.84–3.38)	0.141
Type of chemotherapy regimen		
Paclitaxel vs. docetaxel	1.48 (0.66–3.32)	0.337
PIV		
high vs. low	2.93 (1.40–6.10)	**0.004**
MLR		
high vs. low	2.39 (1.18–4.85)	**0.015**
PLR		
high vs. low	2.43 (1.15–5.13)	**0.020**
NLR		
high vs. low	1.79 (0.88–3.64)	0.105
**Multivariable analysis**	**HR (95% CI)**	***p***
Number of metastatic sites		
≥3 vs. <3	3.78 (1.50–9.52)	0.005
Visceral metastases		
yes vs. no	1.73 (0.72–4.15)	0.217
Previous trastuzumab		
yes vs. no	4.29 (1.85–9.96)	<0.001
PIV		
high vs. low	2.34 (0.99–5.51)	0.052

The *p* value is indicated in bold numbers when statistically significant. * Defined positive if >1% of tumor cells express hormone receptors. Abbreviations: HR hormone receptors; PIV Pan Immune Inflammation Value; MLR monocyte to lymphocyte ratio; PLR Platelet to lymphocyte ratio; NLR neutrophil to lymphocyte ratio.

**Table 3:** Univariate and multivariable Cox proportional hazards model for OS.

Univariate analysis	HR (95% CI)	*p*
AGE		
per 1 year	1.02 (0.98–1.06)	0.374
≥50 vs. <50	2.01 (0.73–5.59)	0.179
HR status *		
Positive vs. negative	0.93 (0.37–2.34)	0.874
*De novo* advanced disease		
yes vs. no	0.76 (0.30–1.91)	0.561
Number of metastatic sites		
≥3 vs. <3	2.57 (1.02–6.49)	**0.045**
Visceral metastases		
yes vs. no	2.46 (0.95–6.41)	**0.065**
Brain metastases		
yes vs. no	4.02 (1.40–11.51)	**0.010**
Type of chemotherapy regimen		
Paclitaxel vs. docetaxel	1.23 (0.41–3.73)	0.714
Previous trastuzumab		
yes vs. no	1.80 (0.75–4.33)	0.190
Previous anthracyclines		
yes vs. no	0.90 (0.37–2.17)	0.811
Previous taxanes		
yes vs. no	1.66 (0.69–4.00)	0.263
PIV		
high vs. low	9.48 (2.69–33.44)	**<0.001**
MLR		
high vs. low	2.82 (1.16–6.85)	**0.022**
PLR		
high vs. low	1.65 (0.63–4.35)	0.308
NLR		
high vs. low	2.65 (0.96–7.30)	0.060
**Multivariable analysis**	**HR (95% CI)**	***p***
Number of metastatic sites		
≥3 vs. <3	1.86 (0.66–5.24)	0.239
Visceral metastases		
yes vs. no	1.37 (0.44–4.32)	0.588
Brain metastases		
yes vs. no	1.44 (0.46–4.53)	0.536
PIV		
high vs. low	7.96 (2.18–29.09)	**0.002**

The *p* value is indicated in bold numbers when statistically significant. * Defined positive if >1% of tumor cells express hormone receptors. Abbreviations: HR hormone receptors; PIV Pan Immune Inflammation Value; NLR neutrophil to lymphocyte ratio; MLR monocyte to lymphocyte ratio; PLR Platelet to lymphocyte ratio.

## Data Availability

The datasets generated and/or analyzed during the current study are available from the corresponding author on reasonable request.

## Supplementary Materials

The following are available online at https://www.mdpi.com/article/10.3390/cancers13081964/s1, Table S1—Patient characteristics according to PIV category; Table S2—Association between PIV and other peripheral blood parameters (as dichotomous variables); Table S3—multivariable Cox proportional hazards model for PFS including NLR, MRL, or PLR; Table S4—multivariable Cox proportional hazards model for OS including and NLR, MLR or PRL; Table S5—PIV as a dichotomous variable (high vs. low) in patients with long vs. short PFS (≥24 months vs. <24 months, respectively); Figure S1—Association between peripheral blood parameters (as continuous variables); Figure S2—PIV as a continuous variable in patients with long vs. short PFS (≥24 months vs. <24 months, respectively).
