# Supplementary material for: The Pan-Immune-Inflammation-Value Predicts the Survival of Patients with Human Epidermal Growth Factor Receptor 2 (HER2)—Positive Advanced Breast Cancer Treated with First-Line Taxane-Trastuzumab-Pertuzumab
Source: Cancers (Basel). 2021 Apr 19;13(8):1964. doi: 10.3390/cancers13081964 (PMC8073809; doi:10.3390/cancers13081964)
Supplement: Supplementary file 1 [file cancers-13-01964-s001.zip › cancers-1160482-supplementary.pdf]

SUPPLEMENTARY MATERIAL

Table S1 – Patient characteristics according to PIV category

	**Low PIV (N=28)**	**High PIV (N=29)**	**Total (N=57)**	**p**
AGE				
Median (Range)	53 (36 -78)	54 (27 -77)	53 (26-78)	0.782*
<50	8 (28.6%)	11 (37.9%)	19 (33.3%)	0.458
≥50	20 (71.4%)	18 (62.1%)	38 (66.7%)	
De novo metastatic disease				
no	15 (53.6%)	19 (65.5%)	34 (59.6%)	0.362
yes	13 (46.4%)	10 (34.5%)	23 (40.4%)	
Number of metastatic sites				
≤2	17 (60.7%)	13 (44.8%)	30 (52.6%)	0.234
>2	11 (39.3%)	16 (55.2%)	27 (47.4%)	
Visceral metastases				
no	17 (60.7%)	10 (34.5%)	27 (47.4%)	**0.049**
yes	11 (39.3%)	19 (65.5%)	30 (52.6%)	
Brain metastases				
no	27 (96.4%)	22 (75.9%)	49 (86.0%)	**0.027**
yes	1 (3.6%)	7 (24.1%)	8 (14.0%)	
HR status**				
no	9 (32.1%)	11 (37.9%)	20 (35.1%)	0.650
yes	19 (67.9%)	18 (62.1%)	37 (64.9%)	
Previous trastuzumab				
no	18 (64.3%)	17 (58.6%)	35 (61.4%)	0.663
yes	10 (35.7%)	12 (41.4%)	22 (38.6%)	
Previous anthracyclines				
no	17 (60.7%)	13 (44.8%)	30 (52.6%)	0.234
yes	11 (39.3%)	16 (55.2%)	27 (47.4%)	
Previous taxanes				
no	17 (60.7%)	14 (48.3%)	31 (54.4%)	0.350
yes	11 (39.3%)	15 (51.7%)	26 (45.6%)	
Type of chemotherapy regimen				
Docetaxel	24 (85.7%)	20 (69.0%)	44 (77.2%)	0.135
Paclitaxel	4 (14.3%)	9 (31.0%)	13 (22.8%)	

Data are presented as n (%) except where otherwise noted. The p value of the $\chi 2$ test assessing the association between each characteristic and the PIV category is indicated in the right column of the table, except where otherwise noted.* Wilcoxon test. **Defined positive if >1% of tumor cells express hormone receptors. Abbreviations: HR, hormone receptors; PIV: Pan-Immune - Inflammation Value

Table S2 - Association between PIV and other peripheral blood parameters (as dichotomous variables)

	**Low PIV (N=28)**	**High PIV (N=29)**	**Total (N=57)**	**p**
PLR				
low	19 (67.9%)	9 (31.0%)	28 (49.1%)	**0.006**
high	9 (32.1%)	20 (69.0%)	29 (50.9%)	
MLR				
low	26 (92.9%)	12 (41.4%)	38 (66.7%)	**< 0.001**
high	2 (7.1%)	17 (58.6%)	19 (33.3%)	
NLR				
low	22 (78.6%)	6 (20.7%)	28 (49.1%)	**< 0.001**
high	6 (21.4%)	23 (79.3%)	29 (50.9%)	

Data are presented as n (%). The p value of the $\chi 2$ test assessing the association between each characteristic and the PIV category is indicated in the right column of the table. Abbreviations: PIV: Pan-Immune -Inflammation Value; MLR: monocyte to lymphocyte ratio; PLR: platelet to lymphocyte ratio; NLR: neutrophil to lymphocyte ratio.

Figure S1 - Association between peripheral blood parameters (as continuous variables)

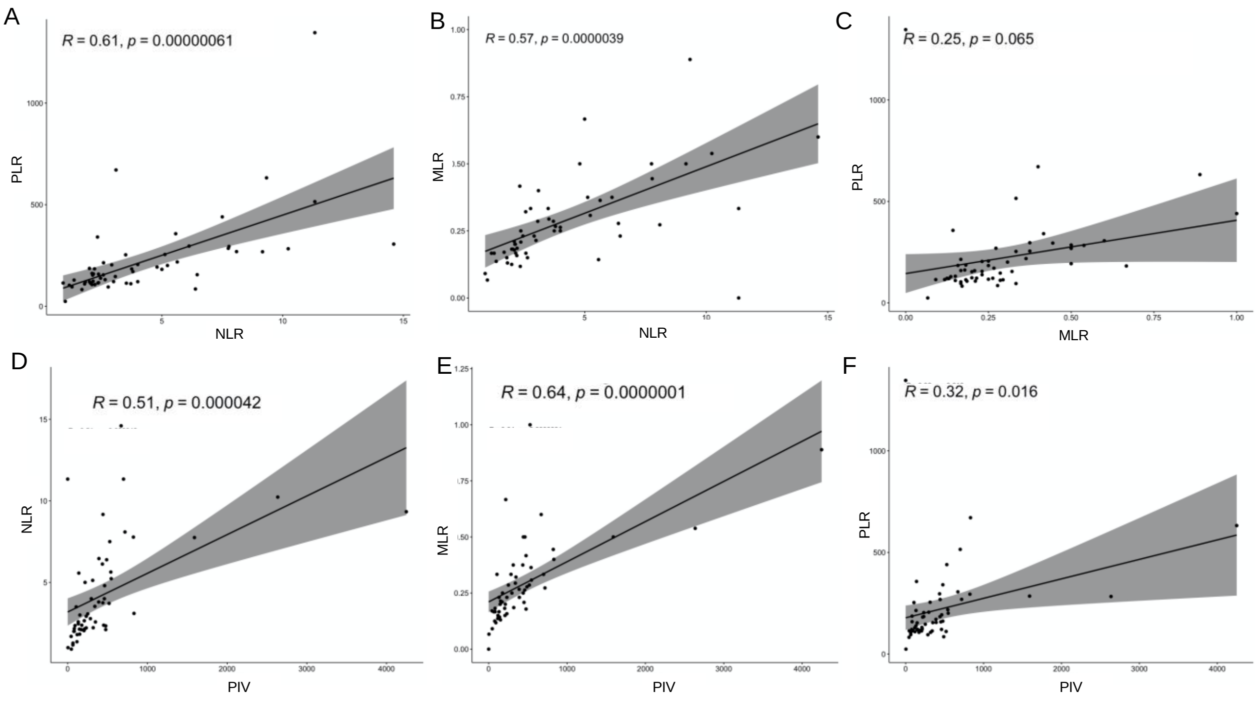

Supplementary Figure 1. Linear correlation between PLR and NLR (A), MLR and NLR (B), PLR and MLR (C); PIV and NLR (D), PIV and MLR (E), PIV and PLR (F) NLR: neutrophil to lymphocyte ratio; MLR: monocyte to lymphocyte ratio; PLR: platelet to lymphocyte ratio.

Table S3 - multivariable Cox proportional hazards model for PFS including PLR, NRL, or MLR

**Multivariable analysis**	**HR (95% CI)**	**p**
Number of metastatic sites		
≥3 vs <3	3.72 (1.46 - 9.48)	**0.006**
Visceral metastases		
yes vs no	2.25 (0.96 - 5.28)	0.063
Previous trastuzumab		
yes vs no	4.42 (1.90 - 0.26)	**<0.001**
PLR		
high vs low	1.93 (0.89 - 4.18)	0.094

The p value is indicated in bold numbers when statistically significant. PLR Platelet to lymphocyte ratio

**Multivariable analysis**	**HR (95% CI)**	**p**
Number of metastatic sites		
≥3 vs <3	3.62 (1.40 - 9.39)	**0.008**
Visceral metastases		
yes vs no	2.39 (1.04 - 5.47)	**0.040**
Previous trastuzumab		

yes vs no	4.58 (1.95 - 10.77)	**<0.001**
MLR		
high vs low	1.80 (0.86 - 3.78)	0.119

The p value is indicated in bold numbers when statistically significant. MLR monocyte to lymphocyte ratio.

**Multivariable analysis**	**HR (95% CI)**	**p**
Number of metastatic sites		
≥3 vs <3	4.11 (1.62 - 10.42)	**0.003**
Visceral metastases		
yes vs no	2.39 (1.04 - 5.52)	**0.041**
Previous trastuzumab		
yes vs no	4.57 (1.89 - 11.04)	**<0.001**
NLR		
high vs low	1.13 (0.53 - 2.42)	0.757

The p value is indicated in bold numbers when statistically significant. NLR neutrophil to lymphocyte ratio

Table S4 - Multivariable Cox proportional hazards model for OS including and PLR, MLR or NRL

**Multivariable analysis**	**HR (95% CI)**	**p**
Number of metastatic sites		
≥3 vs <3	1.99 (0.74 - 5.38)	0.173
Visceral metastases		
yes vs no	1.47 (0.48 - 4.47)	0.502
Brain metastases		
yes vs no	2.81 (0.90 - 8.76)	0.075
PLR		
high vs low	1.16 (0.41 - 3.30)	0.778

The p value is indicated in bold numbers when statistically significant. PLR Platelet to lymphocyte ratio

**Multivariable analysis**	**HR (95% CI)**	**p**
Number of metastatic sites		
≥3 vs <3	1.79 (0.64 - 4.98)	0.265
Visceral metastases		
yes vs no	1.63 (0.54 - 4.92)	0.390
Brain metastases		
yes vs no	2.12 (0.66 - 6.84)	0.210
MLR		
high vs low	2.26 (0.88 - 5.84)	0.091

The p value is indicated in bold numbers when statistically significant. MLR monocyte to lymphocyte ratio.

**Multivariable analysis**	**HR (95% CI)**	**p**
Number of metastatic sites		
≥3 vs <3	1.82 (0.65 - 5.09)	0.254
Visceral metastases		

yes vs no	1.45 (0.47 - 4.44)	0.520
Brain metastases		
yes vs no	2.39 (0.74 - 7.70)	0.145
NLR		
high vs low	1.78 (0.60 - 5.33)	0.302

The p value is indicated in bold numbers when statistically significant. NLR neutrophil to lymphocyte ratio

Figure S2 – PIV as a continuous variable in patients with long vs. short PFS (≥ 24 months vs. <24 months, respectively)

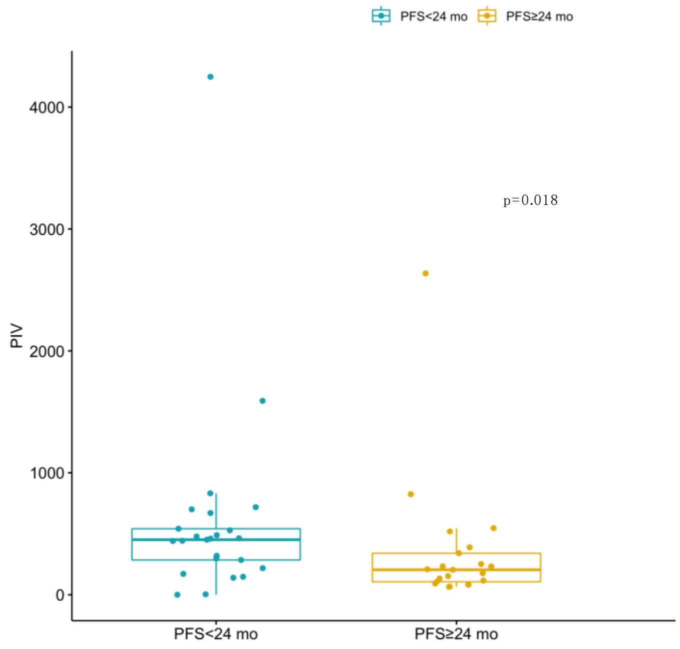

Table S5 – PIV as a dichotomous variable (high vs. low) in patients with long vs. short PFS (≥ 24 months vs. <24 months, respectively)

	PFS ≥ 24 months (n=21)	PFS < 24 months (n=25)	Total (N=46)	p
PIV				0.004*
low	15 (71.4)	6 (24.0)	21 (45.7)	
high	6 (28.6)	19 (76.0)	25 (54.3)	

Long responder patients were those with a PFS of at least 24 months. Patient with a follow up shorter than 24 months were excluded from the analysis. Data are presented as n (%). * $\chi 2$ test Abbreviations: PIV: Pan-Immune -Inflammation Value.
